# Novel Design and Application of High-NA Fiber Imaging Bundles for In Vivo Brain Imaging with Two-Photon Scanning Fluorescence Microscopy

**DOI:** 10.1021/acsami.2c22985

**Published:** 2023-03-07

**Authors:** Łukasz Bijoch, Urszula Włodkowska, Rafał Kasztelanic, Monika Pawłowska, Dariusz Pysz, Leszek Kaczmarek, Radek Lapkiewicz, Ryszard Buczyński, Rafał Czajkowski

**Affiliations:** † BRAINCITY, Nencki Institute of Experimental Biology PAS, Pasteura 3, 02-093 Warszawa, Poland; ‡ Nencki Institute of Experimental Biology PAS, Pasteura 3, 02-093 Warszawa, Poland; § Faculty of Physics, University of Warsaw, Pasteura 5, 02-093 Warsaw, Poland; ∥ Institute of Microelectronics and Photonics, Lukasiewicz Research Network, Al. Lotników 32/46, 02-668 Warsaw, Poland

**Keywords:** in vivo two-photon microscopy, fiber imaging bundles, fluorescence, brain imaging, genetically encoded calcium indicator, green fluorescence protein

## Abstract

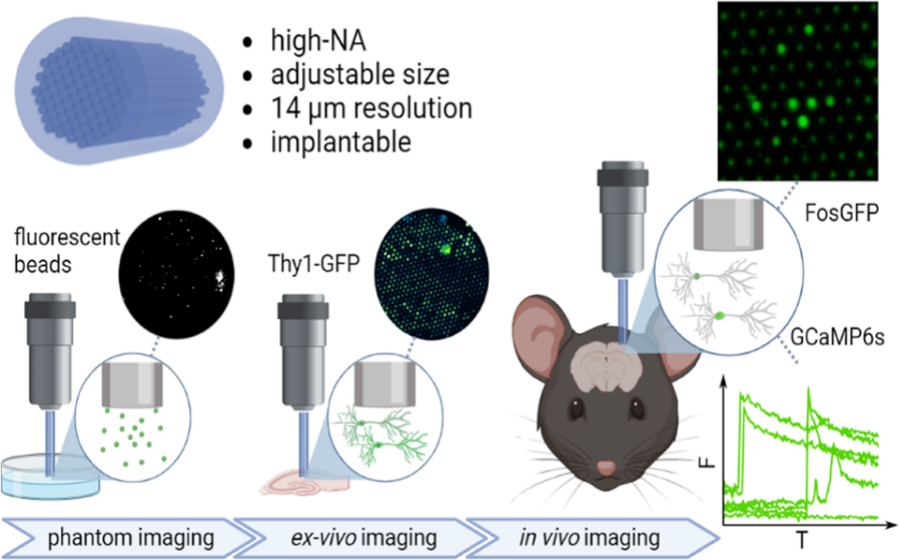

Here, we provide experimental verification supporting the use of short-section imaging bundles for two-photon microscopy imaging of the mouse brain. The 8 mm long bundle is made of a pair of heavy-metal oxide glasses with a refractive index contrast of 0.38 to ensure a high numerical aperture NA = 1.15. The bundle is composed of 825 multimode cores, ordered in a hexagonal lattice with a pixel size of 14 μm and a total diameter of 914 μm. We demonstrate successful imaging through custom-made bundles with 14 μm resolution. As the input, we used a 910 nm Ti-sapphire laser with 140 fs pulse and a peak power of 9 × 10^4^ W. The excitation beam and fluorescent image were transferred through the fiber imaging bundle. As test samples, we used 1 μm green fluorescent latex beads, ex vivo hippocampal neurons expressing green fluorescent protein and cortical neurons in vivo expressing the fluorescent reporter GCaMP6s or immediate early gene Fos fluorescent reporter. This system can be used for minimal-invasive in vivo imaging of the cerebral cortex, hippocampus, or deep brain areas as a part of a tabletop system or an implantable setup. It is a low-cost solution, easy to integrate and operate for high-throughput experiments.

## Introduction

Imaging a living brain on all levels of its morphological complexity and physiological activity is one of the greatest challenges in biomedical research.^1^ This task can be now addressed with a variety of new techniques based on several different physical phenomena. Among them, there are functional magnetic resonance, photoacoustic imaging,^2,3^ and micropositron emission tomography.^4^ These techniques dominate the imaging in the field of cancer research and angiogenesis and have made inroads into neuroimaging.^5^

Two-photon (2P) imaging^6,7^ plays an important role in studies of brain cell morphology^8,9^ and in monitoring neuronal activity.^10−12^ 2P imaging is ideal for biological observations as it avoids phototoxic effects that can occur in fragile brain tissue,^13^ minimizes photobleaching, and allows imaging even in the presence of light scattering. The 2P absorption peak for the most common genetically encoded protein markers is easily achievable by commercially available Ti-sapphire femtosecond lasers.^14,15^ In such studies, head-fixed animals are placed under a microscope, usually lightly anesthetized^16^ or awake but with limited movement, for example, on a floating or rotating stage.^17^ To successfully study biologically relevant phenomena in freely moving animals, lightweight, minimally invasive 2P implantable scanning microscopes have been developed that allow unrestrained movement.^18−22^ Notably, the latest development of low-cost portable 2P laser scanning microscopy (2PLSM)^23^ offers superior image quality with minimum inconvenience to the animal.

A major limitation of all aforementioned techniques is the poor optical access to structures located deep in the brain. Photoacoustic microscopy is limited to approx. 3 mm depth without compromising resolution.^24^ With standard objectives, 2PLSM is restricted only to imaging near-surface areas (up to 1 mm depth) due to the relatively inefficient collection of emission light. It is therefore necessary to design and produce implantable probes that reliably extend the range of imaging techniques while causing minimal damage to the surrounding tissue. Solutions for deep-brain imaging are usually based on gradient index lenses (GRINs) that offer good collection efficacy^25,26^ at the cost of relatively rigid design and high price.

An alternative method for minimally invasive brain imaging is the use of optical fibers. Due to the very limited diameter and unlimited length of operation, they allow imaging of deep brain structures with 2D scanning. Use of fiber probes for 2PLSM requires custom-made fibers since a high numerical aperture (NA) is required to efficiently collect fluorescent signals and dedicated dispersion is needed to deliver ultrashort pulses into the excitation area.

A single optical fiber has been reported to collect 2P images of a single neuron in the cerebral cortex of mice.^27,28^ Currently, fluorescence microendoscopy (FME),^25^ fiber-optic confocal microscopy,^29,30^ and 2P FME^18,19,25,26,31−33^ are the most commonly used fiber-optic fluorescence imaging modalities. For all these techniques, optical fibers provide reduction in the size of the microscope and a flexible design of the probe for delivery of the excitation light. However, regular single-mode fibers (SMFs) have limited ability for efficient excitation beam delivery and fluorescence signal collection.^34^

Many of the limitations of single-mode optical fibers in endoscopic imaging can be circumvented by using multimode optical fibers.^35−38^ Fiber imaging bundles (FIBs) which consist of hundreds of cores are commonly used for typical in vivo imaging applications. FIBs provide direct image transmission where each individual fiber core serves as a single imaging pixel. Notably, the transported image is not affected by distortions caused by bending or twisting of the optical fiber as long as intercore coupling does not degrade the transferred image. FIBs can be used for imaging with or without scanning systems,^39,40^ and they can be incorporated in more sophisticated imaging systems that use confocal^29,39,41^ or structured light illumination approaches.^42−44^ To date, several microscopic imaging demonstrations using FIBs, including deep-brain imaging of a living animal, have been described.^34,45^

Chronic in vivo imaging of neuronal populations of a particular genetic identity is of great interest to neuroscientists as certain psychiatric disorders (e.g., drug dependence, schizophrenia, and so forth) affect unique cell types.^34^ Novel genetic engineering techniques enable the expression of fluorescent genetically encoded calcium indicators (GECIs) specifically in neurons with known genetic identity. This allows us to study the activity of those cells by imaging calcium influx to neurons which corresponds to action potentials.^46^ Another method for imaging of a particular set of neurons is using immediate early gene (IEG) expression to label neurons undergoing plastic changes, during, for example, learning and memory or addiction formation. In both cases, resolution in the range of 20 μm (the size of neuronal soma) is usually sufficient to monitor the desired biological effect.^47^

Here, we designed and manufactured custom FIBs and tested them in order to image biologically relevant samples using a standard tabletop 2P microscope setup. The custom-made cores of high NA and 14 μm pitch allowed for efficient image collection, and no degradation of the femtosecond laser pulse was observed.

Initial approximations of biological material were carried out by imaging fluorescent beads embedded in agarose. The 2P effect was preserved in the imaging bundle to an extent that allows fluorescent excitation of the sample. Next, we confirmed the applicability of the bundles for a fluorescently labeled biological material using brain slices with neurons expressing genetically encoded green fluorescent protein (GFP). Finally, we successfully used the optical layout for live 2P in vivo imaging of intracellular Ca^2+^ changes and the FOS protein reporter in the mouse cortex. With FIBs, we achieved cellular resolution of imaging with a high signal-to-noise ratio. Our FIB design provides substantial advantages for deep microscopic studies of the living brain, including unlimited depth, functionally relevant resolution, high signal-to-noise ratio, and low cost of production.

## Results and Discussion

### Development of FIB

Two types of glass used in separate previous studies were taken into consideration during the development of optical bundles. For the core, we chose a material with a high refractive index, an in-house synthesized, lead-bismuth-galate glass termed PBG08 with the chemical composition as follows (mol %): 40% SiO_2_, 30% PbO, 10% Bi_2_O_3_, 13% Ga_2_O_3_, and 7% CdO.^48^ Basic thermo-physical parameters of the PBG08 glass are presented in Table 1. For the cladding, glass with the lowest possible refractive index was selected. We used borosilicate-type glass composed in the SiO_2_–B_2_O_3_–Al_2_O_3_–Li_2_O–Na_2_O–K_2_O system. The purpose of modifying the glass composition was to maintain a low refractive index while thermally matching the PBG08 glass. This approach ensured that they could be drawn together on the optical tower. We also ensured low-crystallization susceptibility as several thermal processes are required to fabricate the imaging bundles. Finally, as the best fit for the needs of imaging bundle fabrication, we selected in-house synthesized glass, UV710, with the chemical composition as follows (mol %): 53% SiO_2_, 28% B_2_O_3_, 1.5% Al_2_O_3_, 5% Li_2_O, 5% Na_2_O, and 7.5% K_2_O. Basic thermo-physical parameters of the UV710 glass are presented in Table 1.

**Table 1:** Thermo-Physical Parameters of the Glass: α—Linear Thermal Expansion (20 ÷ 450 °C Range), DSP—Dilatometric Softening Point, *T*_g_—Transition Temperature, *T*_z_—Ovalization Point, *T*_k_—Sphere Point, and *T*_pk_—Hemisphere Point

	glass type	
parameters	PBG08	UV710
α (10^–6^K^–1^)	80.1	77.8
DSP (°C)	503	524
temp (°C)		
*T*_g_ log η = 13.4	478	487
*T*_z_ log η = 9.0	540	590
*T*_k_ log η = 6.0	595	680
*T*_pk_ log η = 4.0	660	720

**Figure 1:**
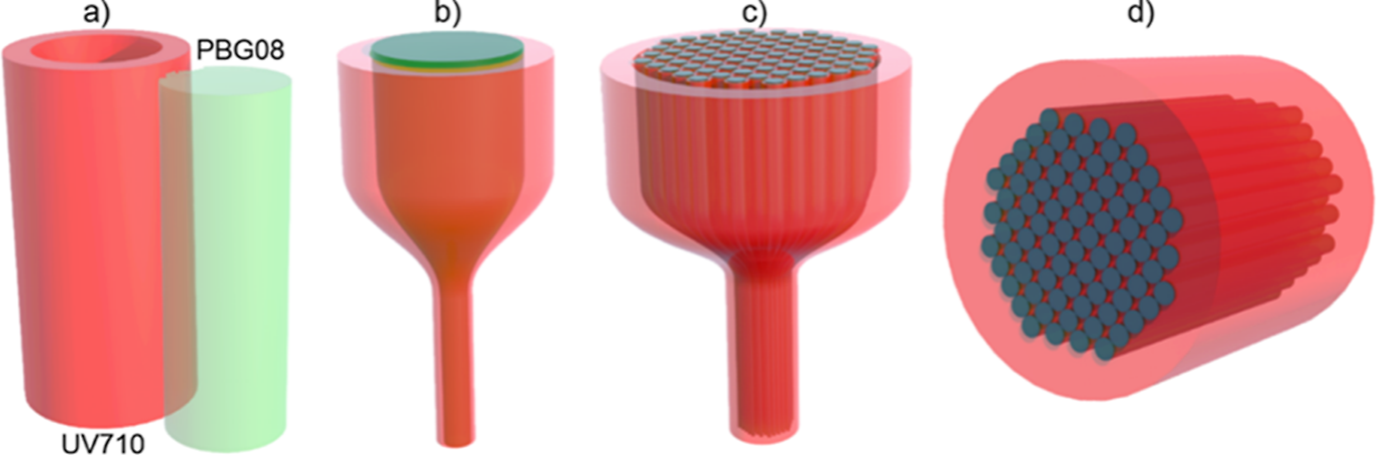
Schematic of the stack-and-draw process for FIB fabrication: (a) rod and capillary preparation, (b) drawing of a single-pixel preform, (c) assembly and drawing the preform, and (d) final optical bundle after cutting and polishing.

To develop the FIB elements, we used a modified standard technique for fabricating the step-index fibers.^49^ This technique consists of several steps shown in Figure 1. First, a PBG08 glass rod and a UV710 glass capillary are prepared (Figure 1a). The inner diameter of the capillary is slightly larger than the diameter of the rod so that the rod can be inserted into the capillary. The outer diameter of the capillary is chosen to finally achieve the designed separation between the individual cores in the optical bundle. Next, after inserting the rod into the capillary, both are drawn on the optical fiber drawing tower and scaled down to obtain a rod with a diameter of about 400 μm (Figure 1b). Then, rods fabricated in this procedure are inserted into a new capillary made of low-index glass (UV710) to form the final preform. The rods form a hexagonal structure where each rod performs the role of a single “pixel” in the optical bundle. Finally, the FIB preform is drawn on the optical fiber drawing tower and scaled down (Figure 1c). This approach enables FIBs to be obtained with varying diameters and pitch between each “pixel” from the same preform. Furthermore, it ensures that the individual “pixels” in a particular FIB have precisely the same optical properties. After cutting the drawn-out fiber to the desired length and after polishing the ends, the final FIB was obtained (Figure 1d).

An important factor that may influence the amount of crosstalk between neighboring “pixels” is the diffusion. It may cause blurring of the sharp boundary between the core and the cladding material which in consequence leads to degradation of the contrast of registered images. Therefore, the drawing process is carried out at a relatively low temperature and slow speed to minimize diffusion.

**Figure 2:**
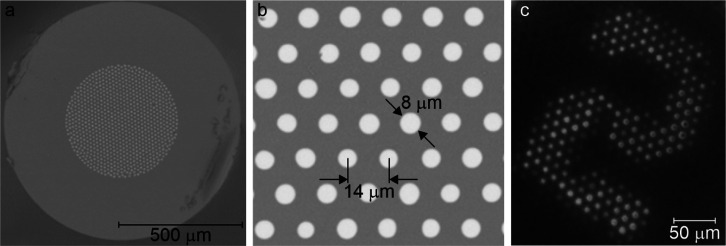
Fabricated FIB: (a) scanning electron microscopy (SEM) image of the whole imaging bundle, (b) enlarged area with cores, and (c) example of imaging of the 1951 USAF resolution test.

The FIB fabrication method described here allows for strict control of the size of the cores and the distance between them. Notably, both of these parameters may easily be adjusted for specific applications. To proceed with performance tests, we produced FIBs with an 8 μm core diameter located at a 14 μm interval (Figure 2). We predicted that this layout would allow error-free detection of objects with a diameter of 20 μm, corresponding to the size of a neuronal soma (Figure S1).

**Figure 3:**
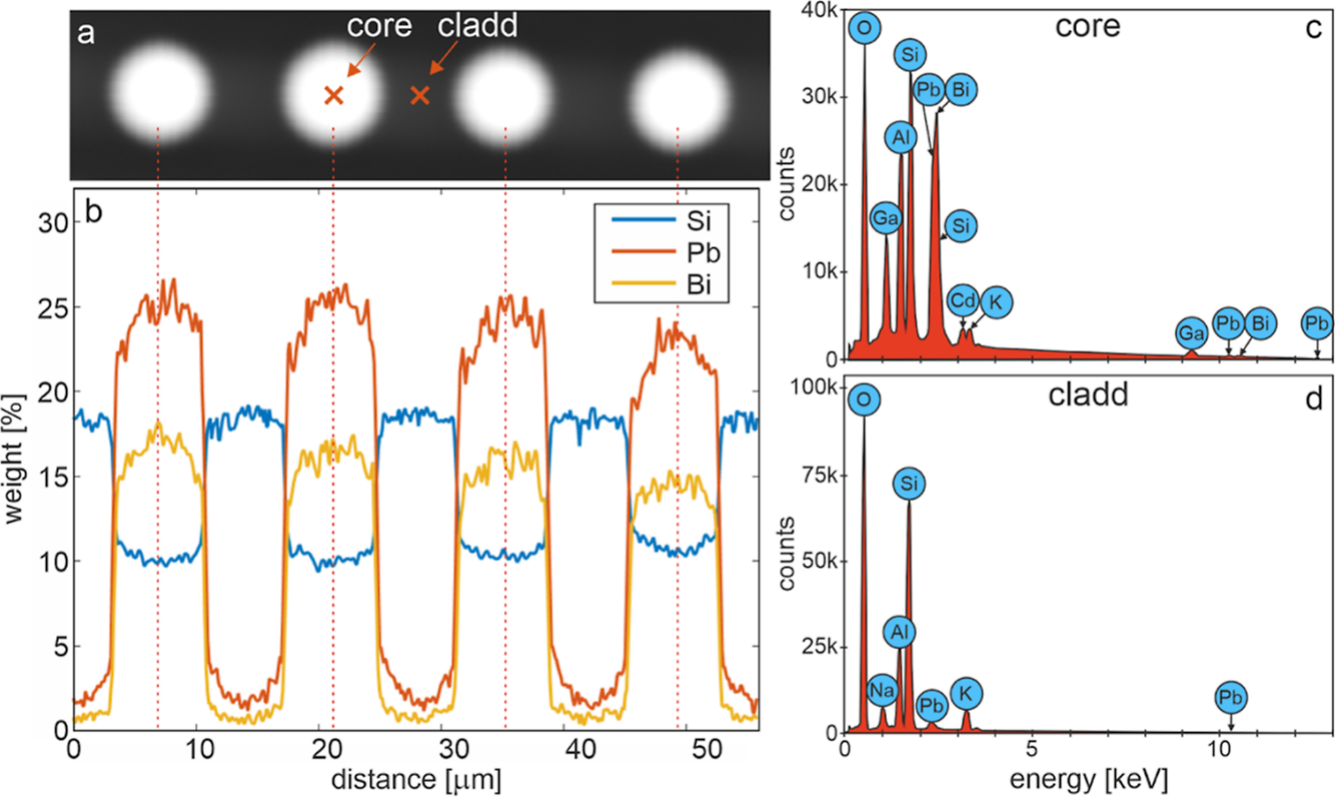
Results of EDS measurement: (a) SEM image of the FIB fragment, (b) content (weight in %) of the basic elements that compose the FIB, (c) elemental content in the core area, and (d) elemental content in the cladding area.

Energy-dispersive X-ray spectroscopy (EDS) measurements (Figure 3) demonstrate that the distribution of individual elements and thus the distribution of the refractive index were as expected and diffusion does not play a significant role.

The fabricated FIB had a diameter of 914 μm, and the diameter of the core area was only 450 μm. This was not a limiting factor for the imaging of latex beads and ex vivo fixed tissue. However, for in vivo imaging, it was necessary to reduce the diameter of the outer coat to minimize tissue damage. This was achieved by etching the glass using a 5% hydrofluoric acid solution. The use of a low concentration of acid required about 3 h for the reduction of the FIB diameter to 500 μm and resulted in good-quality FIB side surfaces. A longer section of FIB (about 20 cm) was etched and then cut and polished into 8 mm sections, which were used in further experiments.

### Optical Properties of FIB

**Figure 4:**
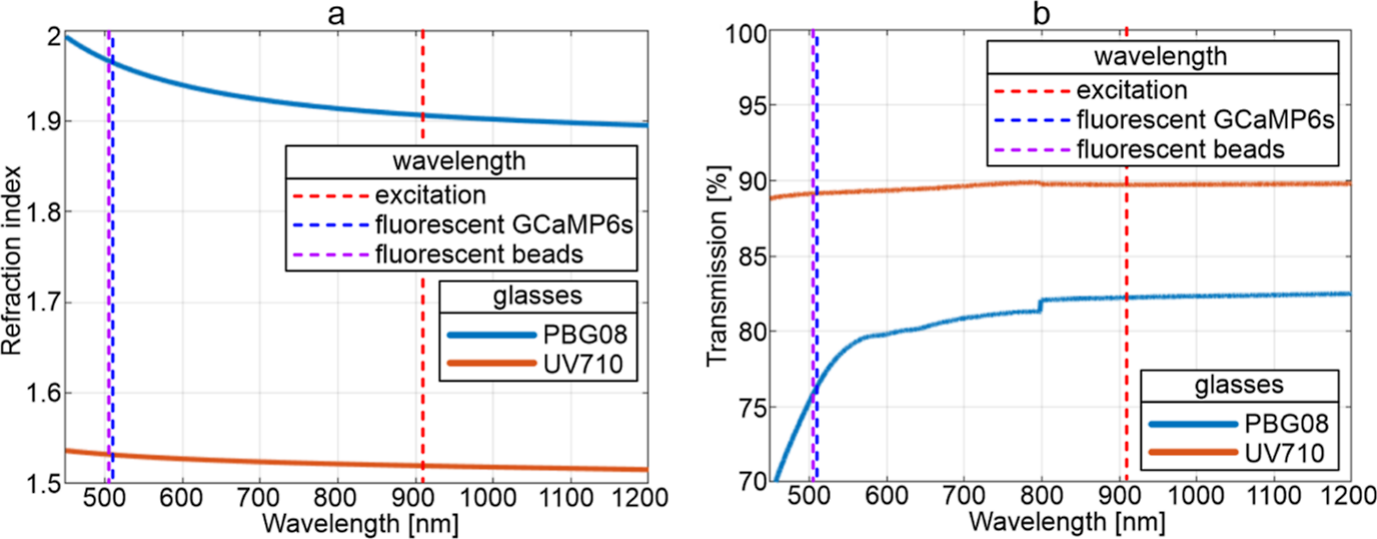
PBG08 and UV710 glass properties: (a) refractive indices measured using a Michelson interferometer and (b) light transmission for an 8 mm length sample measured using a Bruker IFS 113 V Fourier transform infrared spectrometer.

The bulk refractive indices for the PBG08 and UV710 glass are described using the Sellmeier coefficients presented in Table 2 and Figure 4a. Both types of glass were characterized by high light transmission in the range from 480 nm to near-infrared (Figure 4b). The fabricated FIB had a very high numerical aperture (NA = 1.15) for an excitation light length of 910 nm. The high NA reduces optical crosstalk between neighboring cores and consequently improves the contrast and quality of the image.^50^ For the fabricated FIB where the 8 μm diameter cores are 14 μm apart, the crosstalk was negligible.

**Table 2:** Sellmeier Coefficients of the Glass

	glass type	
Sellmeier coefficients	PBG08	UV710
B1	2.011881	1.2
B2	0.546732	0.105503
B3	1.394886	1.3
C1 (μm^2^)	0.015375	0.007039
C2 (μm^2^)	0.063552	0.023827
C3 (μm^2^)	141.654046	113.881899

The high NA also affects the efficiency of light collection. The collection efficiency (η) for a single core if the FIB is located in a medium with refractive index n_0_ and collecting light from a planar fluorescent source with area *A*_s_ is given as^51,52^
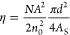
where *d* denotes the core diameter and *A*_s_ is a function of the distance *z* between the fluorescent plane and the fiber surface
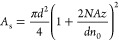


**Figure 5:**
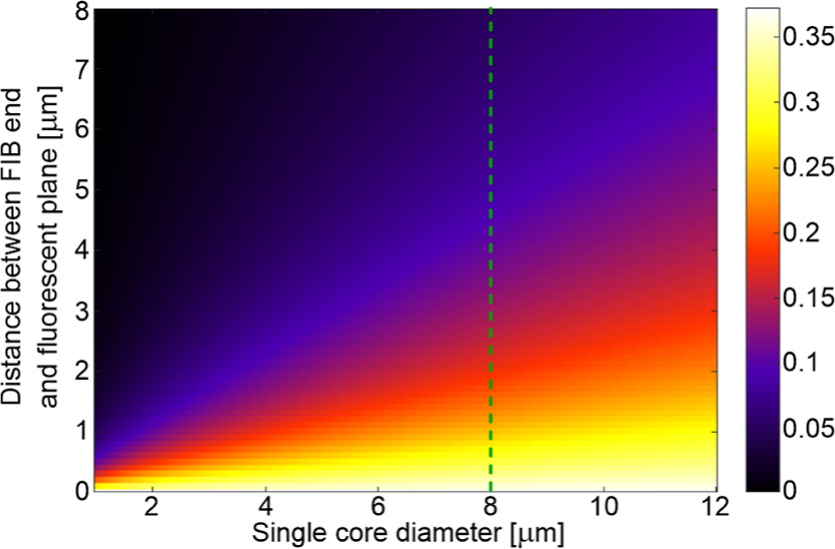
Dependence of fluorescence light collection on the diameter of a single core and the distance between the end of the FIB and the imaged plane. The green dashed line represents the core diameter of the fabricated FIB.

η values calculated for FIB with various single core diameter *d* and distance between FIB end and fluorescence plane *z* are presented in Figure 5. This relationship shows that, for a given core diameter, light collection efficiency decreases rapidly as the distance from the end of the FIB increases. The maximum efficiency of above 37% is achieved at the end of the FIB. At 2 μm from the end of the FIB, the efficiency is already 2 times lower. As such, this type of FIB is particularly suitable for imaging objects located at the layer directly adhering to the end of the FIB while minimizing the impact from layers located at greater distances.

**Figure 6:**
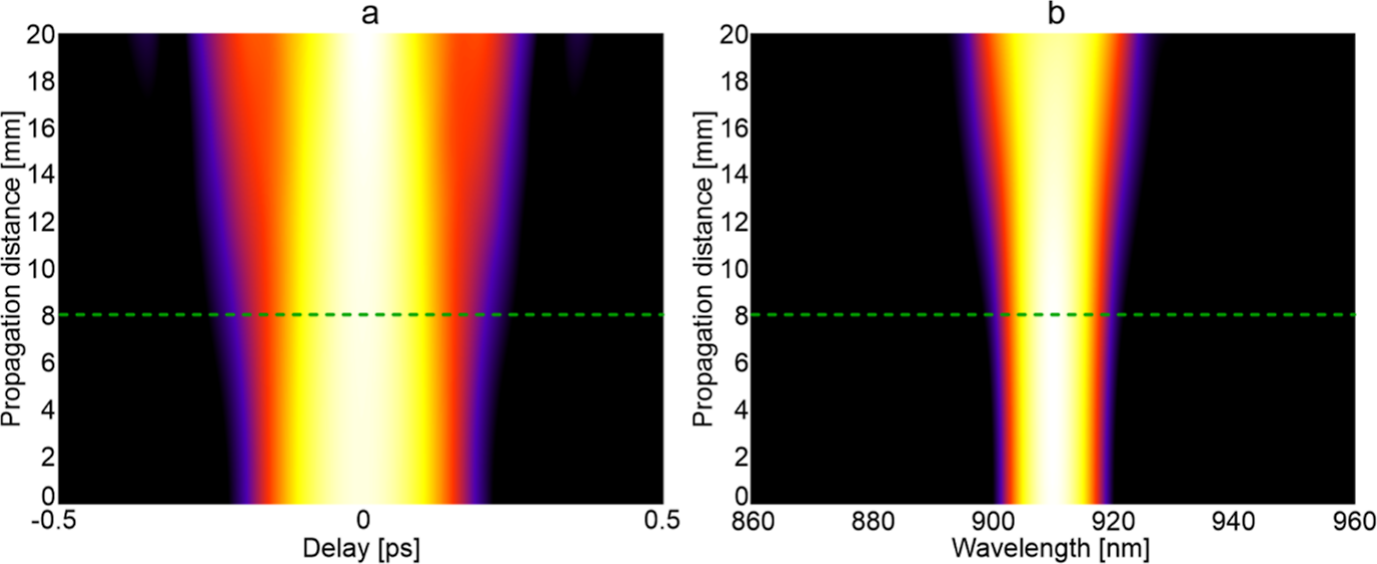
Evolution of the 140 fs pulse in a single core made from PBG08 glass taking into account the dispersion and nonlinear effects: (a) temporal and (b) spectral. The green dashed line indicates the propagation length in the experimental setup.

With two-photon excitation, the material of the FIB should not significantly affect the excitation pulse. For the FIB described here, the PBG08 core glass had a nonlinearity of 4.3 × 10^–19^ m^2^/W. The experiments used a Ti-sapphire laser with a wavelength of 910 nm, 80 MHz repetition rate, and a pulse duration of 140 fs. The average excitation power used was 10 mW, which corresponds to about 893 W for a single pulse. Nonlinear simulations show that the pulse slightly broadens in the time domain (Figure 6a) and the spectral domain (Figure 6b). The initial 140 fs pulse expands to 142 fs for propagation along the 8 mm distance and about 180 fs along the 20 mm distance. This suggests that the FIB described here can be used for 2P imaging at greater depths, reaching several centimeters.

### 2PLSM and Confocal Imaging

**Figure 7:**
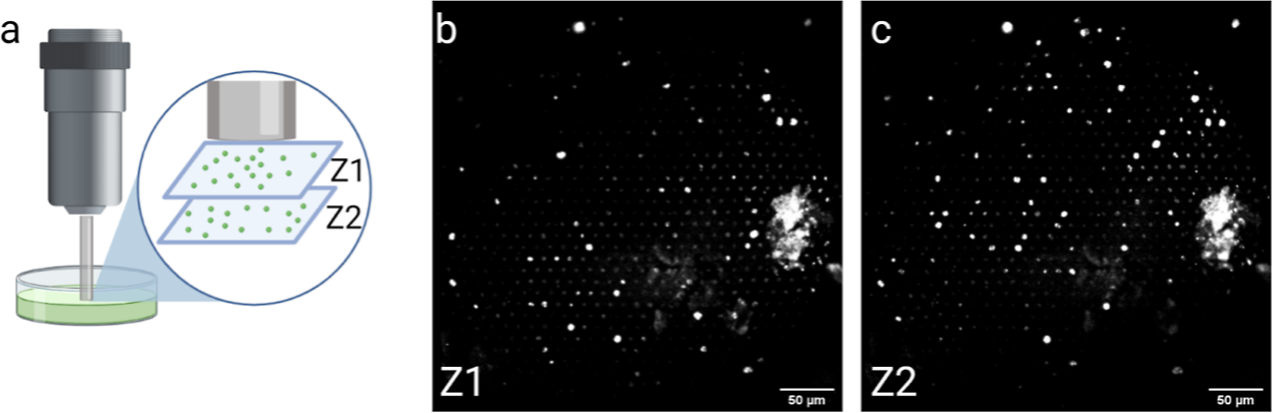
2P imaging of fluorescent latex beads using a combination of FIB and 10× Plan-Neofluar objective lens. (a) Experimental scheme. Fluorescent beads (1 μm diameter) were suspended in 1% agarose, and scans were performed after positioning the optical bundle on two planes separated by 100 μm. (b,c) Single-scan images of the back aperture of the optical bundle at Z1 and Z2 planes. Scale bar, 50 μm.

To test whether the manufactured optical bundles are useful for biological applications, we first obtained control images of fluorescent beads suspended in agarose gel. Agarose phantoms are widely used as a substitute for brain tissue due to their similar mechanical properties.^53^ Aside from providing early insights into the optical features of the system, such phantom helps to develop a proper implantation protocol and to minimize insertion damage. We followed a previously described procedure used for testing in vivo optical setups^54^ without unnecessary harm to the experimental animals. We were able to detect fluorescent signals from individual beads, each coming from a separate core of the bundle (Figure 7b,c). Moving the bundle in the *Z* axis by 100 μm changed the imaged pattern, suggesting that the observed signal was not an artifact. Images of fluorescent latex beads obtained via the optical bundles have therefore shown that it is possible to utilize custom-made optical bundles in combination with the in vivo 2P optical setup. Moreover, the application is optimized for fluorescent reporters that visualize targets of the size similar to the facets of the optical bundle.

**Figure 8:**
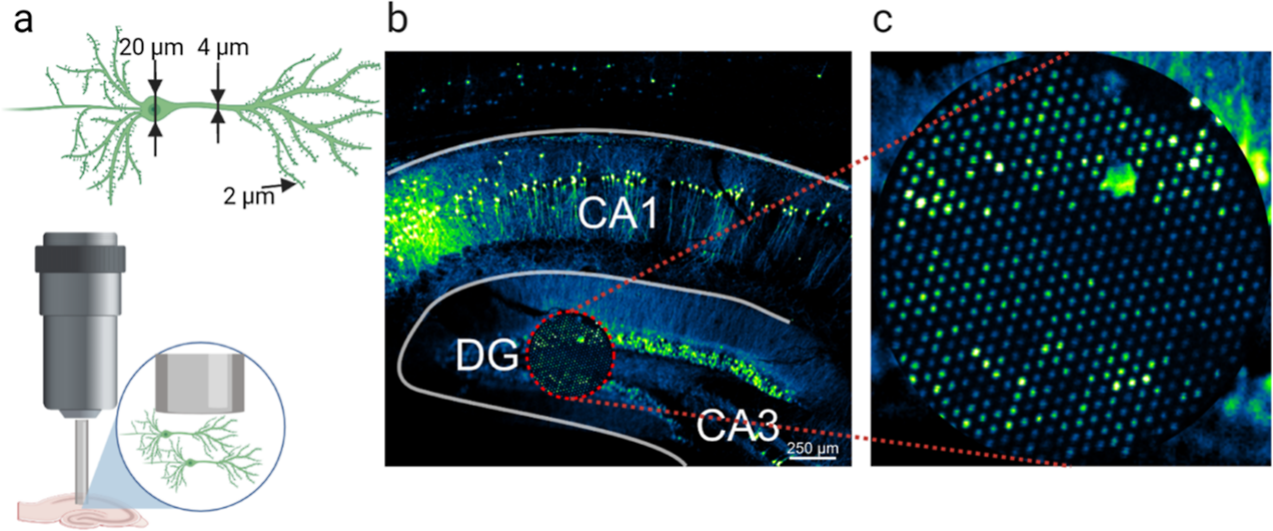
Confocal imaging of hippocampal Thy1-GFP neurons using a combination of FIB and 10× Plan-Neofluar objective lens. (a) Experimental scheme and neuronal fluorochrome distribution. (b) Confocal image of Thy1-GFP neurons in the hippocampal slice. The area covered by FIB is indicated by dashed circle (c). Zoomed image of the FIB-imaged area. Scale bar, 250 μm.

After confirming the overall applicability of the imaging setup, we selected a widely used fluorescent reporter, GFP, for further tests using a biological material. We first expressed the reporter from the Thy-1 promoter in a transgenic mouse model. In this line, GFP is abundantly present in a relatively sparse population of excitatory neurons.^9,55^ This allows monitoring the somatic presence of GFP and also studying the detailed morphology of each individual neuron in vivo and in fixed tissue. We prepared fixed slices of Thy1-GFP hippocampi and mounted them under the confocal microscope (Figure 8a, see the Experimental Section for details). Using the FIB approach, we were able to observe cell bodies, but as expected, the morphological details of the neurons (2–4 μm) were beyond resolution (Figure 8c).

**Figure 9:**
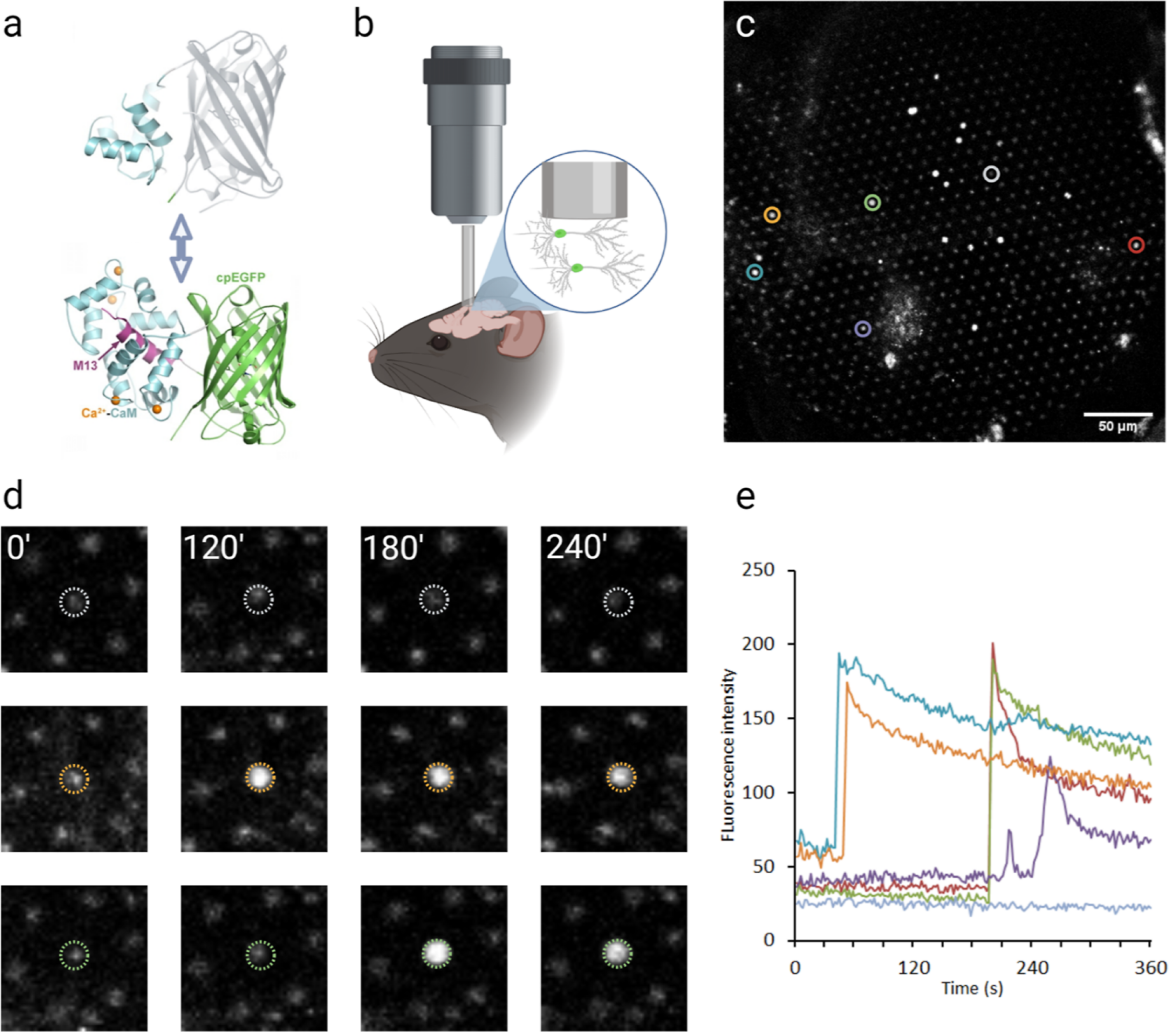
Time-lapse in vivo 2P imaging of GECI in mouse RS with a combination of FIB and 10× Plan-Neofluar objective lens. (a) Mechanism of GECI reporting. Upon binding of cytoplasmic Ca^2+^ ions by the calmodulin domain, a conformational change allows GFP to absorb photons and emit fluorescence. (b) Experimental scheme. The Ca^2+^ indicator was expressed from the viral vector, and the FIB was implanted over the viral injection site. Imaging of GECI-positive neurons was performed at 0.5 Hz for 360 s. (c) Overview of the imaging plane taken at 360 s with six ROIs representing individual neurons indicated by circles. (d) Examples of fluorescence levels for selected ROIs at four time points (0, 120, 180, and 240 s). (e) Time traces of average fluorescence changes for ROIs selected in (c). Trace colors correspond to circles in (c,d). Scale bar, 50 μm.

For the in vivo 2P approach, we first decided to utilize GCaMP6s, a GECI. This fluorescent protein fills the entire neuronal cytoplasm and changes its fluorescence upon binding of free cytoplasmic Ca^2+^ ions (Figure 9a). GCaMP6s is used widely to monitor neuronal activity since calcium influx from extracellular space and internal stores follows action potential generation.^11^ Expressing the reporter in a rigorously controlled recombinant adeno-associated virus (rAAV) system resulted in sparse labeling of individual neurons. Importantly, each neuron was detected in a separate core of the optical bundle (Figure 9c). We monitored spontaneous changes in neuronal activity in a lightly anesthetized mouse. We successfully imaged the retrosplenial cortex (RS), a cortical area receiving and processing sensory inputs and also initiating signaling to lower brain structures (top–down processing). We predicted that it would still be partially active during isoflurane anesthesia. As expected, we observed spontaneous activity in a subset of RS neurons (Figure 9d,e). The relevant parameters of the recorded GCaMP6s signal were consistent with previous observations.^46^ Specifically, the d*F*/*F*_0_ value calculated for individual regions of interest (ROIs) ranged between 1.8 and 4.7 (av d*F*/*F*_0_ = 2.82 ± 1.15, see the Supporting Information). Moreover, analyzing the time-variable signals allowed us to confirm experimentally that in this imaging modality, no cross-talk between neighboring cores was observed.

**Figure 10:**
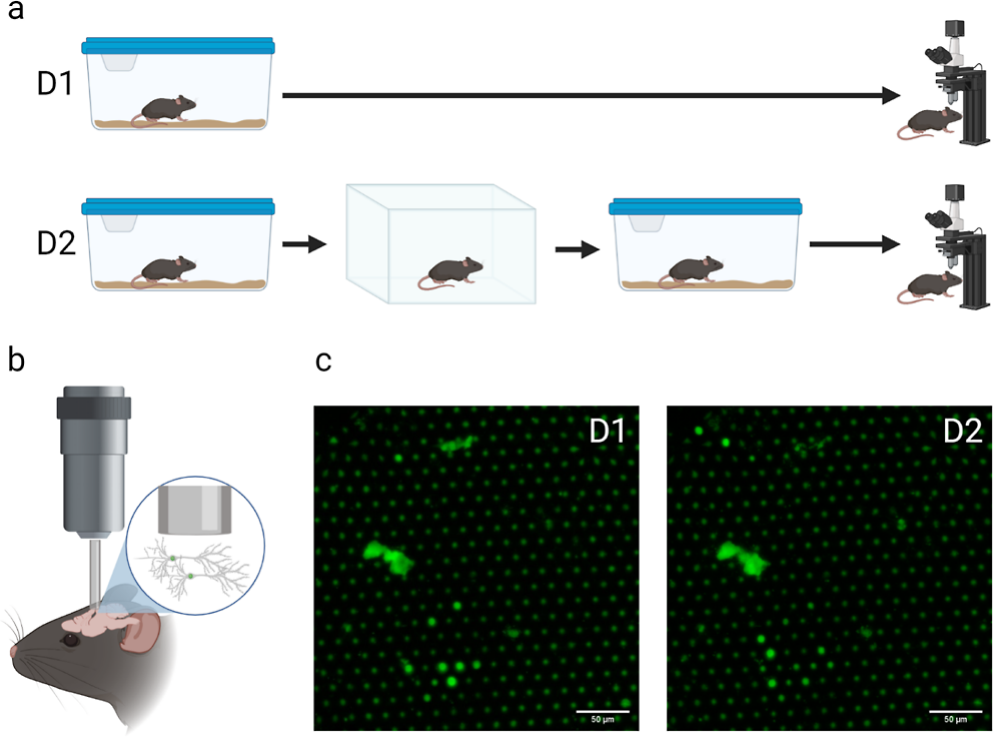
Chronic in vivo 2P imaging of Fos activity patterns in mouse RS with a combination of FIB and 10× Plan-Neofluar objective lens. (a) Experimental scheme. On day 1, the mouse was removed from the home cage and the FOS-GFP reporter was imaged. On day 2, the mouse was subjected to 15 min exploration in a novel box and returned to the home cage. 90 min after the onset of exploration, another image of the same area of RS was acquired. (b) Diagram of the FIB implantation and reporter protein cellular location. (c) Corresponding images of the same area of RS after home cage exploration (D1) and novel box exploration (D2).

Another potentially useful function of the proposed 2P imaging approach with FIB is the in vivo monitoring of IEG activity. IEGs form a diverse group of genes encoding proteins that are often utilized as cellular markers of neuronal activation and reorganization (neuronal plasticity).^47^ One of the IEGs encodes transcription factor protein FOS that accumulates rapidly in the cell nucleus, reaching peak concentration 90 min after activation and then decaying to basal levels within 6 h.^56^ Individual FOS-positive neurons create a brain expression pattern that is unique for a given behavioral stimulus. We used a well-known reporter of Fos gene activity that expresses a FOS-GFP fusion protein, closely matching spatial and temporal regulation of native FOS protein.^10,57^ We compared Fos gene expression pattern in the RS between two behavioral conditions: the standard cage and a novel environment (Figure 10a). Both behavioral stimuli were separated by a 24 h interval, allowing the FOS protein level to reset. As expected, we observed different FOS-GFP patterns for both conditions. This observation demonstrates that the FIBs described here can be effectively used for tracking cell nucleus-targeted fluorescent reporters.

### Methodological Considerations

In this proof-of-concept study, we developed multicore MMFs. We predicted that the performance of multicore MMF would be superior to single-core MMF or multicore SMF. In the case of single-core MMF, to obtain a readable image, it is necessary to use advanced techniques that require interference with the optical system and/or the use of complex algorithms. When a light wave couples to single-core MMF, numerous spatial modes can be guided. These modes can be used for imaging purposes, but wave distortion arising from mode dispersion needs to be corrected.^58^ Several solutions have been proposed to overcome this distortion problem. A wave-front shaping technique has been developed to enable transmission imaging using single-core MMF.^36^ Also, distortion could be eliminated using the speckle imaging method and demonstrated using wide-field endoscopic imaging.^35^ However, in these methods, transmission matrix calculation, image reconstruction processes, or/and scanning mechanism at the distal end of the fiber^19,59−61^ are required to obtain transmission or fluorescence images. In addition, single-core MMF-based systems are highly sensitive to bending or twisting due to the variations in the transmission matrix.

For FIB based on single-mode cores, a common problem is the low efficiency of sample illumination and collection of the excited fluorescence signal.^62^ The key to improving these parameters is to increase NA, which, while maintaining the size of single cores, results in guiding more spatial mods. Commercial FIBs are manufactured from a limited variety of available glasses, limiting the NA values of FBs to around 0.56.^63^ Higher NA values are critical for reducing optical crosstalk between the fibers and hence improving the overall imaging performance. This is especially important in the case of fluorescence imaging. Fluorescence emission obtained from the excitation of tissues has a Lambertian profile of emission and limited intensity to avoid overheating of the tissues. Therefore, the large NA of FIB is necessary to transfer measurable signals via the FB without crosstalk. Currently, the best FIBs dedicated to fluorescent imaging have pixel sizes larger than 3 μm and NA values smaller than 0.40.^28^ The FIB we fabricated with NA = 1.15, 8 μm core, and 14 μm pitch reduces crosstalk between neighboring “pixels” to practically zero. Recently, we also reported the fabrication and characterization of flexible FIB with the core diameter = 1.6 μm, core-to-core distance = 2.3 μm, and NA = 0.53.^64^ These parameters for both FIBs imply better collection efficiency of fluorescent radiation than other FIBs.

The specific design of FIBs used in this study was based on the assumption that cell-size objects would be successfully detected with confocal microscopy and 2PLSM (Figure S1a). Indeed, in the case of the GCaMP6s reporter that fills the entire soma, individual neurons were unequivocally resolved and time-lapse measurement of fluorescence changes was possible for each cell. In the case of Thy1-GFP, the somata were also visible, but dendritic trees and axons (also strongly labeled with the reporter) were beyond the resolution of the FIB. FOS-GFP, a reporter that localizes to the cell nucleus, was also detected in vivo. In this case, the diameter of the labeled nuclei (approx. 10 μm) was on the threshold of the FIB resolution, and we could not exclude the possibility that certain neurons may be omitted from the scan (Figure S1b). For the future studies, FIBs made from UV710 and PBG08 glasses can be linearly scaled down to a size where the core diameter is about 3 μm and the distance between the centers of the cores is approx. 5.2 μm.

A slight modification to the optical setup—introducing a spatial light modulator to the excitation path—would enable 2PLSM through our FIB with diffraction-limited resolution in 3D limited only by FIB’s NA and without the image pixelation due to the structure of FIB.^65^ Remarkably, in such an approach, the focused excitation spot can be scanned in 3D at the distal end of the FIB by controlling the wavefront of the excitation beam before it passes the FIB. The wavefront optimization can be performed in situ relying on the nonlinearity of two-photon excited fluorescence as a feedback mechanism. Our FIB with its exceptionally high NA is particularly attractive for this approach.

## Conclusions

In this work, we designed, manufactured, and experimentally verified a new type of fiber imaging bundles for use with in vivo 2PLSM. It is characterized by a very high NA = 1.15 and a core size tailored to the imaging of cortical neurons. As a proof-of-concept, we produced a system composed of 8 mm long custom-made imaging bundles with a diameter of 914 μm, reduced to 500 μm after chemical etching. These imagining bundles have negligible influence on the input pulse parameters. Due to dispersion, the pulse length increased by 1.43%, remaining within the range necessary for 2P excitation of biologically relevant samples.

We demonstrated the applicability of this technique using a model system and further verified experimentally through in vitro and in vivo brain imaging. Initial observations using fluorescent latex beads embedded in agarose demonstrated the overall compatibility of the FIB with the typical 2P microscopy setup. In vivo recordings from GECI- and Fos-GFP-expressing mice provided proof for the sufficiency of both spatial and temporal resolution to distinguish the transcriptional or metabolic activity of single neurons. This confirmed our initial assumption regarding the fiber diameter and spacing within the bundle.

One of the key advantages of the proposed system is its high versatility and low production cost. The size of individual facets and the diameter of the bundle core can be easily scaled during the production process. The final length of the implantable bundle can be adjusted with high accuracy according to the needs of the experimenter. Importantly, the length of the FIBs used in the proof-of-concept is suitable for imaging deep brain structures like the amygdala. It is also possible to combine FIBs of different lengths and diameters to, for example, simultaneously image multiple brain regions. This approach is now possible not only with tabletop 2P setups but also with the recently developed low-cost high-performance in vivo system, capable of scanning surfaces up to 5 mm^2^.^23^

## Experimental Section

### Animals

For the in vivo experiment, we used a C57/Bl6 mouse or FosGFP line.^57^ For the slice imaging, we used the Thy1-GFP line.^55^ Both transgenic lines were maintained in the C57/Bl6 background. Animals were kept on a 12 h light/dark cycle. Food and water were available ad libitum. All procedures were approved by the 1st Local Ethical Committee in Warsaw (LEC protocol 905/2019).

### Thy1-GFP Slice Imaging

Thy1-GFP mouse was perfused transcardially with phosphate-buffered saline (PBS) enriched with heparin for 10 min and then with 4% paraformaldehyde in PBS for 5 min. The brain was sliced coronally into 200 μm sections using the Leica VT 1000S vibratome and kept in PBS at 4 °C. A slice with visible hippocampal formation was placed under the upright confocal microscope (Zeiss Axio Examiner.A1 with STEDYCON system by Aberrior Instruments GmbH). FIB was held with bulldog clamps (F.S.T 18374-43) attached to the manual micromanipulator (Marzhauser 00-42-101-0000) and then placed at 90° at the brain slice. During imaging of each region, the surface of the FIB was imaged first. Then, after removing the FIB away from the field of view, the image of the brain slices was acquired.

### General Surgical Procedures

A modified, previously described procedure^16^ was used. The anesthesia was induced with 5% isoflurane in a chamber and maintained in 2–1% isoflurane in the anesthesia mask. Additional analgesia was provided by injection of Butomidor (3.3 mg/kg). Standard stereotactic surgical procedures were used. To prevent inflammation, animals were treated with Baytril (2.5 mg/kg) 5 days after surgery. They were also injected with Tolfedine (2 mg/kg) for 2 days to provide analgesia. Mice were allowed to recover for 2 weeks.

### rAAV Injection

After sterilizing with betadine and 70% ethanol, a circular patch of skin (1 cm diameter) was carefully removed from the top of the skull. The target coordinate was marked using stereotactic references (bregma: AP 3.0 mm and ML -1.0 mm). The craniotomy was made with a dental drill (bur diameter 1 mm). An AAV vector (serotype AAV1) was used to express GCaMP6s (Addgene no. 100843-AAV1). Recombinant AAV (titer 1 × 10^13^ mg/mL) was injected into the retrosplenial cortex (bregma: DV 1.5 mm). Each animal received a total volume of 0.4 μL using a 35G needle with a 10 μL NanoFil syringe assembled on a UMP3 pump (WPI, Sarasota, FL, USA). Injection took 10 min, and we waited for 10 additional minutes before retracting the syringe. The injection site was stabilized with gelfoam and saline.

### FIB Implantation

Custom-length FIBs (8 mm length) were used. FIBs were cleaned and sterilized in 70% ethanol and then placed in a holder mounted on the stereotaxic arm. FIB was placed above the AAV injection side 2.5 mm into the tissue. This step was done slowly to prevent tissue compression. FIB was stabilized with Loctite 420 adhesive and dental acrylics to expose approx. 5 mm of the bundle length. Next, two small titanium bars were attached to the dental acrylic cap to stabilize animals during imaging sessions. The protruding tip of the optical bundle was covered with a protective plastic cap affixed to the dental cement with a Kwik-Cast (WPI) silicone sealant.

### 2P Microscope

A Zeiss 7 MultiPhoton InVivo microscope was used with EC-Plan-Neofluar 10× objective with NA = 0.1. Several modifications were made to accommodate the whole animal under the objective lenses. A custom made stage with a heating pad and a head mount was installed. A Coherent Chameleon 2P laser was used with a tuning range of 690–1040 nm and a pulse width of 140 fs. The laser was tuned to 910 nm for fluorescent beads and GCaMP6s excitation.

### 2P Imaging of Fluorescent Beads

Fluorescent beads (Sigma-Aldrich L1030) 1 μm in diameter were used with single-photon excitation and emission peaks at λ_ex_ = ∼470 nm and λ_em_ = ∼505 nm. Beads were sonicated and suspended in a warm solution of 1% agarose in distilled water at a dilution of 1:1000. The solution was cast in a 3 cm Petri dish and mounted under the microscope. FIB was inserted 1,5 mm deep into the agarose beads solution using a micromanipulator (Marzhauser 00-42-101-0000). The microscope objective was focused on the back aperture of the bundle. An *XY* scan was acquired at a resolution of 1024 by 1024 pixels (0.415 μm per pixel). The bundle was then lowered by 100 μm in the *Z* axis, and another scan was taken.

### 2P Imaging of the GCaMP6s Fluorescent Reporter

4 weeks after surgery, the animal was lightly anesthetized with isoflurane (induction 5%, maintenance 1%) and placed under a custom made microscope mount. The silicone cover was removed from the FIB, and the objective lens was focused on the back aperture of the bundle. Continuous *XY* scan was then performed at a frequency of 0.5 Hz for 360 s, resulting in 180 frames. The resolution was set to 512 by 512 pixels (0.692 μm per pixel). Time traces for each facet of the FIB were extracted using a custom-written Python script with Napari library for image processing.^61^ Briefly, a hexagonal network of coordinates covering the FIB facets was created, and for each point, the signal was averaged over a circle. In total, 789 traces were extracted (see the Supporting Information). d*F*/*F*_0_ values were calculated for individual ROIs. Average fluorescence 30 s prior to the onset of response was used as F_0_.

### 2P Imaging of the FOS-GFP Fluorescent Reporter

On the 1st day of the experiment, the animal was removed from its home cage, lightly anesthetized with isoflurane (induction 5%, maintenance 1%) and placed under the custom-made microscope mount. The silicone cover was removed from the FIB, and the objective lens was focused on the back aperture of the bundle. *XY* scan was then performed at a resolution of 512 by 512 pixels (0.692 μm per pixel). The animal was then released into the home cage for recovery. After 24 h, the animal was placed for 15 min in a novel environment (square box). After 90 min, the imaging procedure was repeated.

## Supporting Information Available

Time traces for all analyzed facets of the FIB with rows corresponding to the analyzed fiber facets and columns corresponding to time series frames (XLSX)
Schematic detection of biologically relevant objects with FIB (PDF)

## Abbreviations

2P: two photon
2PLSM: two-photon laser scanning microscopy
EDS: energy-dispersive X-ray spectroscopy
FIB: fiber imaging bundle
FME: fluorescence microendoscopy
GECI: genetically encoded calcium indicator
GFP: green fluorescent protein
GRIN: gradient-index
IEG: immediate early gene
LEC: Local Ethical Committee
MMF: multimode fiber
NA: numeric aperture
OB: optical bundle
rAAV: recombinant adeno-associated virus
ROI: region of interest
RS: retrosplenial
SEM: scanning electron microscopy
SMF: single-mode fiber
