# Supplementary material for: Novel Design and Application of High-NA Fiber Imaging Bundles for In Vivo Brain Imaging with Two-Photon Scanning Fluorescence Microscopy
Source: ACS Appl Mater Interfaces. 2023 Mar 7;15(10):12831–41. doi: 10.1021/acsami.2c22985 (PMC10020965; doi:10.1021/acsami.2c22985)
Supplement: Supplementary file 2 — am2c22985_si_002.pdf [file am2c22985_si_002.pdf]

Supporting information

Novel design and application of high-NA fiber imaging bundles for *in vivo* brain imaging with two-photon scanning fluorescence microscopy

*Łukasz Bijoch, Urszula Włodkowska, Rafał Kasztelanic, Monika Pawłowska, Dariusz Pysz, Leszek Kaczmarek, Radek Łapkiewicz, Ryszard Buczyński, and Rafał Czajkowski**

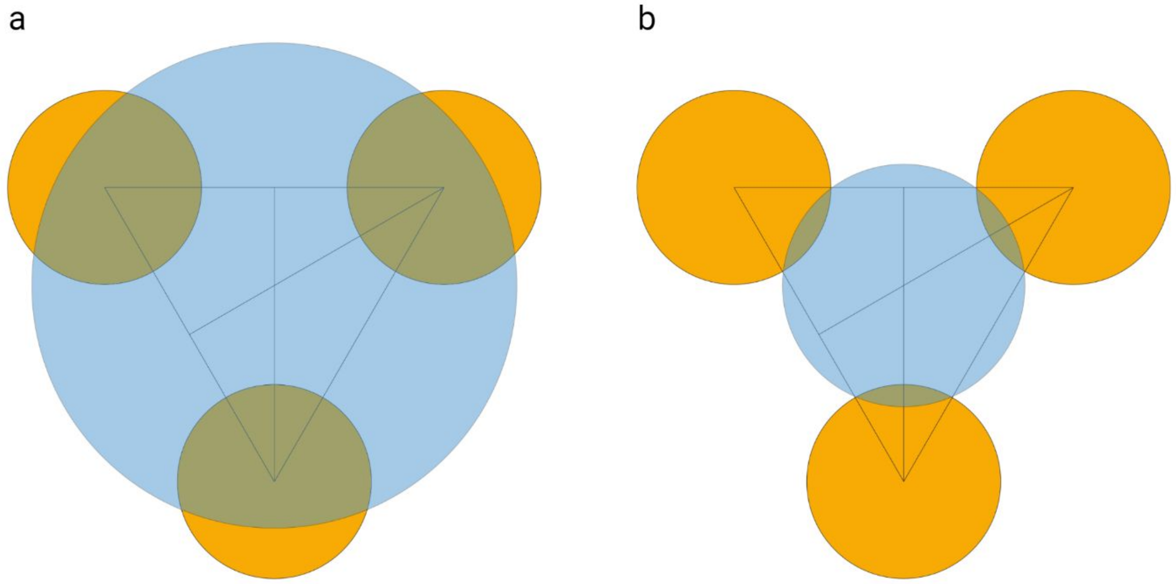

Figure S1 Detection of object with diameter of 20 µm (a) or 10 µm (b) with the FIB.
